# A novel approach for maintaining alignment of sigmoid vaginoplasty anastomosis of urogenital sinus repair complicated with iatrogenic rectovaginal fistula: case report

**DOI:** 10.1093/jscr/rjaf466

**Published:** 2025-07-01

**Authors:** Abubakr H Mossa, Omer H Mossa, Samir Al-Ahmad, Hilal Matta, Khalid Khalfan Sabet, Muhammad Eyad Ba'ath

**Affiliations:** College of Medicine, University of Sharjah, University City, PO Box 27272, Sharjah, United Arab Emirates; University Hospital Sharjah, University City, PO Box 27272, Sharjah, United Arab Emirates; University Hospital Sharjah, University City, PO Box 27272, Sharjah, United Arab Emirates; Tawwam Hospital, Khalifa Bin Zayed Road, Al-Maqam, Al-Ain, United Arab Emirates; University Hospital Sharjah, University City, PO Box 27272, Sharjah, United Arab Emirates; University Hospital Sharjah, University City, PO Box 27272, Sharjah, United Arab Emirates

**Keywords:** urogenital sinus, sigmoid vaginoplasty, rectovaginal fistula, case report

## Abstract

Redo surgeries for persistent urogenital sinus repair are challenging, often requiring innovative techniques to address complications like fistula formation and vaginal stenosis. This case report describes the successful management of a complex redo persistent urogenital sinus repair in a 5-year-old girl. Her initial repair at age 1 resulted in an iatrogenic rectovaginal fistula, severe narrowing of the vagina, and required a sigmoid colostomy and tube colpostomy. At age 5, surgical reconstruction involved excising the stenosed urethra, re-anastomosing it to the introitus, and fistula closure. Due to the fragile and thin-walled vagina, a sigmoid interposition vaginoplasty was performed. Extensive pelvic adhesions made direct suturing impossible, so a Foley catheter was used innovatively as a stent to approximate the vaginal wall to the sigmoid interposition. One year later, the vagina and urethra remain patent, and the child is continent following colostomy closure. The catheter stent technique proved effective in ensuring alignment and patency.

## Introduction

Failure of the separation between the urethra and the vagina during embryonic development results in the rare congenital abnormality of persistent urogenital sinus (PUGS) [1]. PUGS can be isolated or part of more complex congenital syndromes, such as congenital adrenal hyperplasia (CAH) [2]. The arrest in urogenital separation results in a confluence, whether high or low, between the vagina and the urethra forming a common channel leading into a single opening at the introitus [1]. While antenatal ultrasonography helps identify many cases of PUGS by detecting fluid collections such as hydrocolpos, late presentations of recurrent urinary tract infection, urinary incontinence, and dribbling are reported [3, 4]. Surgical correction of PUGS creates separate urinary and genital orifices, which can be technically challenging [5]. Due to the extensive dissection needed, devascularization leading to complications such as fistula formation and vaginal strictures is well recognized [3, 5]. These complications usually require redo surgery, which can be extremely difficult and risky. Inevitably, surgical improvisation is sometimes needed in such scenarios.

## Case report

A 5-year-old girl presented to the pediatric surgery clinic in September 2023. She was born in a low-resource war zone with an abdominopelvic mass, which necessitated the insertion of a tube colpostomy. She was then diagnosed with PUGS and underwent an attempted repair at the age of 1 year. During the first surgery, the vagina was severely dilated, encasing the rectum with very thin walls. Rectal iatrogenic injury was encountered and repaired, and the pull-through was completed. Then, a sigmoid colostomy was created to ensure fecal diversion. The pull-through stenosed, and within a month, she presented with an infected hydrometrocolpos requiring re-insertion of the tube colpostomy. The patient was then lost to follow-up, and the family relocated to the United Arab Emirates. The patient showed dysmorphic features such as a broad nose and hypertelorism with moderate developmental delay and polydactyly. An endocrinology review ruled out the possibility of CAH. A computed tomography (CT) scan of the head and abdomen showed no significant visceral or neurological findings. Full genetic profiling of the patient was not undertaken due to the family's reluctance and financial constraints.

**Figure 1:**
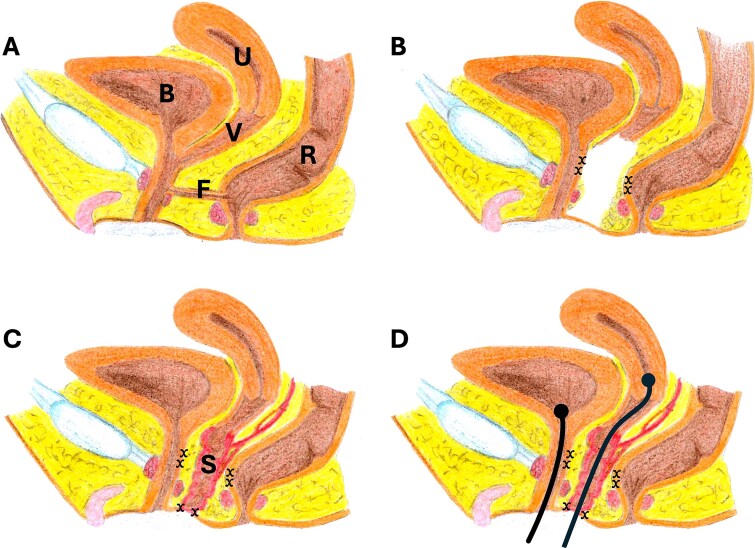
Illustration of the operation steps: (A) the anatomy of the pelvic organs showing the common channel and the fistula (F) location, (B) the fistula was dissected and excised, and the common channel was dissected, (x) indicates the suturing sites, (C) a segment of the sigmoid (with its blood supply and mesentery) was used for sigmoid vaginoplasty, sutured to the skin, (D) urethral catheter and vaginal catheter were placed, the vaginal catheter was applied to overcome the difficulty of suturing the upper vaginal segment with the sigmoid segment (note the absence between the sigmoid segment and the vaginal tube), bladder (B), uterus (U), vagina (V), fistula (F), sigmoid (S).

Examination under general anesthesia showed a normal anus, a single narrow opening at the introitus with scarring. Serial dilatations with Hegar dilators allowed the insertion of a cystoscope. A 3.5 cm-long common channel was seen ending with a well-formed bladder neck and a narrow vaginal confluence immediately below the bladder neck. The vaginal insertion required the use of a small catheter initially, which was then traced by scope. A 3–4 cm-wide rectovaginal fistula was noted with a vaginal opening on the common channel, 1.5 cm above the introitus, and the rectal opening just above the dentate line (Fig. 1A).

Definitive repair was then undertaken. A combined perineal and abdominal approach was utilized. The bladder and the vagina, and the fistula were all cannulated, and then the common channel was dissected. The stenotic urethra was excised, and the vagina was separated from the confluence point. The rectovaginal fistula was closed, with the vaginal side discarded as part of the stenotic urethra/common channel. The residual part of the common channel was utilized as urethra and anastomosed in an orthotopic position to the introitus. The residual vagina was quite short, thin-walled, and fixed due to scarring. The gap between the vagina and the introitus was >6 cm which prevented primary vaginal pull-through (Fig. 1B). Therefore, a sigmoid interposition vaginoplasty was chosen. A loop of sigmoid was isolated on its vascular supply and pulled down. The intention was to suture the proximal end to the lower vaginal opening. Due to the narrow and long pelvic canal and the presence of extensive scarring, this was not possible from either the perineal or abdominal side. The vagina could not be dissected more freely and brought to a higher position in the abdomen safely without compromising its blood supply or risking injury to surrounding structures (Fig. 1C). Due to this, the distal end was anastomosed to the introitus, and a large Foley catheter (24F) was passed through the sigmoid graft and then into the vagina. The balloon was inflated in the already dilated uterus. Pulling the catheter down, the vagina and the sigmoid graft were aligned and approximated. The distal catheter end was secured to the skin. The perineal body was repaired, and the wounds closed (Fig. 1D).

The child recovered well, and the vaginal stent was kept in place for 3 weeks. An examination under anesthesia after three weeks showed a significant narrowing of the vaginal anastomoses that required dilatation. The child was subsequently placed on daily home dilatation. The colostomy was closed 2 months later. A year later, the child had full fecal continence with mild urinary urge incontinence requiring oxybutynin. The vagina remains patent and easily admits 5 Hegar.

## Discussion

We report a challenging case of a redo-surgery of a PUGS repair complicated with adhesions, iatrogenic rectovaginal fistula, and vaginal stenosis. The vaginal tissue fragility and the narrow and long pelvis prevented the conventional end-to-end suturing of the sigmoid interposition to the short and high vaginal tube. Utilizing the catheter as a stent to align the sigmoid and vaginal openings allowed the formation of the anastomosis without suturing the fragile tissue and excessive tissue mobilization. Postoperative outcomes were satisfactory regarding wound healing and continence.

PUGS affects ~6 per 100 000 female births [1]. Its embryological basis highlights the complexity of genitourinary differentiation [1] with diverse clinical presentations. Severe cases may involve hydronephrosis or hydrometrocolpos. Mild forms, however, may remain asymptomatic or exhibit minimal clinical signs [3]. The clinical presentation of our reported case indicates a severe and high confluence form of isolated PUGS.

Different surgical techniques to correct PUGS exist, depending on the confluence level and associated abnormalities [5–7]. Surgical treatment of high-confluence PUGS presents significant challenges, frequently necessitating advanced reconstructive techniques [3, 8]. Most cases with short common channels can be treated with total urogenital mobilization and pull-through vaginoplasty, which entails detaching the vagina from the urogenital sinus and rerouting it to a perineal opening without tension [3, 5, 7]. When the common channel is long (4 cm or more), as in our patient, additional flap techniques are advised to prevent vaginal stenosis and enhance esthetic outcomes [3, 7]. The timing of PUGS is controversial, but there is a trend toward delaying surgical correction to benefit from the role of female sex hormones in vaginal growth [3, 9]. Here, the family was keen on early surgical correction due to the presence of a tube colpostomy and a colostomy.

Utilizing the catheter as a stent was an innovative idea to overcome the unexpectedly weak and fragile vaginal wall. Sutureless anastomotic wound closure has been previously described with multiple methods, such as tissue glues, laser welding, hydrogel patches, and inverted or magnetic rings [10]. This has been implemented in multiple organs, including skin, dental procedures, intestinal anastomoses, and ophthalmic and vascular surgeries [11–14]. These techniques remain experimental, especially in intestinal anastomoses, and have not entered mainstream practice [10]. To our knowledge, this technique has never been used in the context of sigmoid vaginoplasty as in our case.

Close postoperative follow-up is critical to prevent complications and ensure functional and psychological well-being. Ongoing monitoring of continence, urinary retention, recurrent infections, and regular vaginal dilation are mandatory [3]. Long-term outcomes of PUGS repair are underreported in the literature.

## Conclusion

Utilizing a Foley catheter as a trans-anastomotic stent has proven to be a possible solution to overcome tissue fragility and difficulty of access encountered in redoing PUGS repair. Combining sigmoid vaginoplasty and catheter stenting can also reduce the operation time and maintain alignment and patency of the anastomosis.
